# Hidden relationships between metalloproteins unveiled by structural comparison of their metal sites

**DOI:** 10.1038/srep09486

**Published:** 2015-03-30

**Authors:** Yana Valasatava, Claudia Andreini, Antonio Rosato

**Affiliations:** 1 Magnetic Resonance Center (CERM) – University of Florence, Via L. Sacconi 6, 50019 Sesto Fiorentino, Italy; 2 Department of Chemistry – University of Florence, Via della Lastruccia 3, 50019 Sesto Fiorentino, Italy

## Abstract

Metalloproteins account for a substantial fraction of all proteins. They incorporate metal atoms, which are required for their structure and/or function. Here we describe a new computational protocol to systematically compare and classify metal-binding sites on the basis of their structural similarity. These sites are extracted from the MetalPDB database of minimal functional sites (MFSs) in metal-binding biological macromolecules. Structural similarity is measured by the scoring function of the available MetalS^2^ program. Hierarchical clustering was used to organize MFSs into clusters, for each of which a representative MFS was identified. The comparison of all representative MFSs provided a thorough structure-based classification of the sites analyzed. As examples, the application of the proposed computational protocol to all heme-binding proteins and zinc-binding proteins of known structure highlighted the existence of structural subtypes, validated known evolutionary links and shed new light on the occurrence of similar sites in systems at different evolutionary distances. The present approach thus makes available an innovative viewpoint on metalloproteins, where the functionally crucial metal sites effectively lead the discovery of structural and functional relationships in a largely protein-independent manner.

Metal ions are bound to biological macromolecules via coordination bonds. The bonds are formed by the so-called donor atoms, which can belong to either the backbone or side chains/bases of the macromolecule (protein or nucleic acid). Additional donor atoms may belong to non-macromolecular ligands, such as oligopeptides, small organic molecules, anions, water molecules. The metal ion (or cluster of metal ions) together with its donor atoms constitute the metal-binding site. To achieve a satisfactory understanding of the biochemical properties of metal sites through the analysis of 3D structural features it is important to go beyond metal-binding sites by taking into account the surrounding macromolecular environment^1^,^2^,^3^,^4^,^5^,^6^. Altogether, this larger ensemble of atoms defines the minimal environment determining metal function, i.e. the “minimal functional site” (MFS). In practice, we defined an MFS in a metal-macromolecule adduct as the ensemble of atoms containing the metal ion or cofactor, all its ligands and any other atom belonging to a chemical species within 5 Å from a ligand (Supplementary Figure S1)^7^. The MFS describes the local 3D environment around the cofactor, independently of the larger context of the protein fold in which it is embedded. The MetalPDB database, which is derived in an automated manner from the Protein Data Bank (PDB)^8^, collects all known MFSs^9^. Recently, we have developed a computational approach, implemented in the MetalS^2^ program, to quantify the structural similarity of MFSs in metalloproteins^10^.

Structure-based as well as domain-based classifications of protein structures are well established. Resources such as CATH^11^ or SCOP^12^ are able to capture distant relationships between protein domains through the analysis of their 3D structures. They provide the notion of protein superfamily, which is the ensemble of all the protein domains with the same overall structural features. In MetalPDB we exploited such classifications to assign MFSs to so-called equistructural groups^9^. Such groups contain the MFSs that are found in proteins with the same fold and occur at the same position within that fold. This is evaluated by superimposing the entire domain containing the MFS in the protein structures under consideration and then computing the distance between the metal centers. MFSs whose metal centers are within a threshold of 3.5 Å from one another are assigned to the same equistructural group. This approach is simple and intuitive, but can potentially overlook structural variations occurring within each metalloprotein family.

In this work we implemented and evaluated an approach based on the MetalS^2^ program to perform systematic, quantitative comparisons of MFS structures with the final aim of producing a classification of metal sites. This is achieved by organizing MFSs into clusters in such a way that each cluster contains sites that are structurally similar to each other and differ from sites of the other clusters. The resulting classification is independent of the overall metalloprotein fold and can capture the fine structural variability of sites even within the same metalloprotein family. In addition, it provides unbiased indications on relationships between different metalloprotein families harboring the same metal cofactors. This contribution provides an unprecedented approach in bioinorganic structural biology that puts metal sites, the true focus of research in bioinorganic chemistry, at the center of structural analysis. In fact, our new protocol innovatively recombines available algorithms to support out-of-the-box thinking about relationships among metalloproteins. The box we are referring to here is that constituted by the conventional tools based on global sequence or structural domain similarity. The present protocol is not meant to replace this kind of analysis, which has been successfully applied to metalloproteins^13^,^14^,^15^,^16^,^17^,^18^, but to provide an additional, new tool to the portfolio of the structural biologist with an interest in bioinorganic chemistry that has been specifically designed for the distinct challenges of the latter field of research.

We demonstrate the protocol using two test cases, namely heme-binding and zinc-binding proteins. Heme is one of the most abundant and widely used biological metalloporphyrins. As a protein cofactor, heme shuttles electrons between different redox centers in aerobic and anaerobic respiration as well as photosynthesis, or transports and stores O_2_ as with the globins. Furthermore, numerous heme-dependent enzymes are known, which can catalyze both reductive and oxidative chemistry. MetalPDB shows that the iron coordination geometry in heme-containing MFSs is quite constant, being either square pyramidal or octahedral in the vast majority of cases, with four donor atoms out of a maximum of six provided by the porphyrin moiety. This makes it difficult to exploit the features of the iron coordination for functional or structural classification. Zinc proteins are one of the largest groups of metalloproteins within MetalPDB. Estimates of zinc proteomes in various organisms indicated that the amount of genes encoding zinc proteins varies from 4% to 10% of the genome^19^,^20^. Zinc enzymes in which zinc plays a catalytic role are present across all living organisms and constitute the largest share of prokaryotic zinc proteins. The main reason for the selection of zinc as a catalytic cofactor lies in its distinctive chemical properties, which combine Lewis acid strength, lack of redox reactivity, and fast ligand exchange^21^. The coordination geometry of the zinc(II) ion and the number of cysteine ligands can be quite informative on function, both for enzymes^7^,^22^ and non-catalytic systems such as zinc fingers^23^. The application of our newly developed protocol can provide a means to verify structure similarities beyond the first coordination sphere, and their relationship to functional properties.

## Results

### Analysis of equistructural groups of MFSs (first stage)

The present new computational protocol highlights local structure features that may distinguish members within a given metalloprotein family or reveal similarities across different families. To do so, the protocol leverages the organization of sites in equistructural groups (EGs hereafter) that is already provided by the MetalPDB database. These are groups of corresponding sites in the structures of metalloproteins belonging to the same family. Comparisons are first done within EGs, i.e. within metalloprotein families. Then representative MFSs are defined for the various structural subtypes occurring within a family. Finally, representative MFSs are exploited to systematically compare sites across different subtypes and, most importantly, across different metalloprotein families.

**Figure 1:**
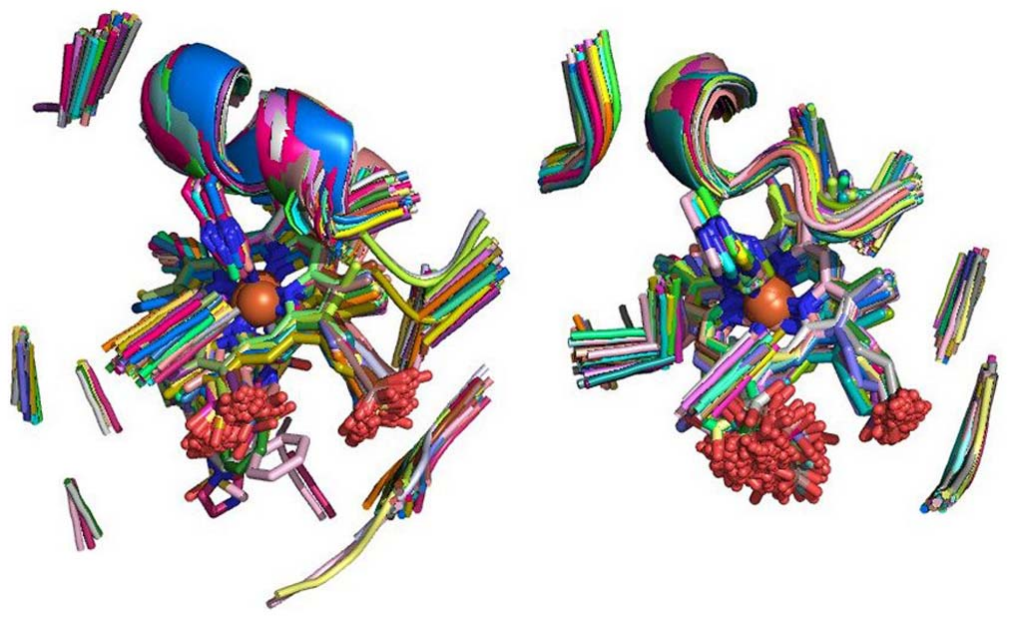
Comparison of the structures of the hMFSs contained in the two major clusters originating from the equistructural group of animal heme-dependent peroxidases. Left: myeloperoxidases and lactoperoxidases; right: prostaglandin synthases.

**Figure 2:**
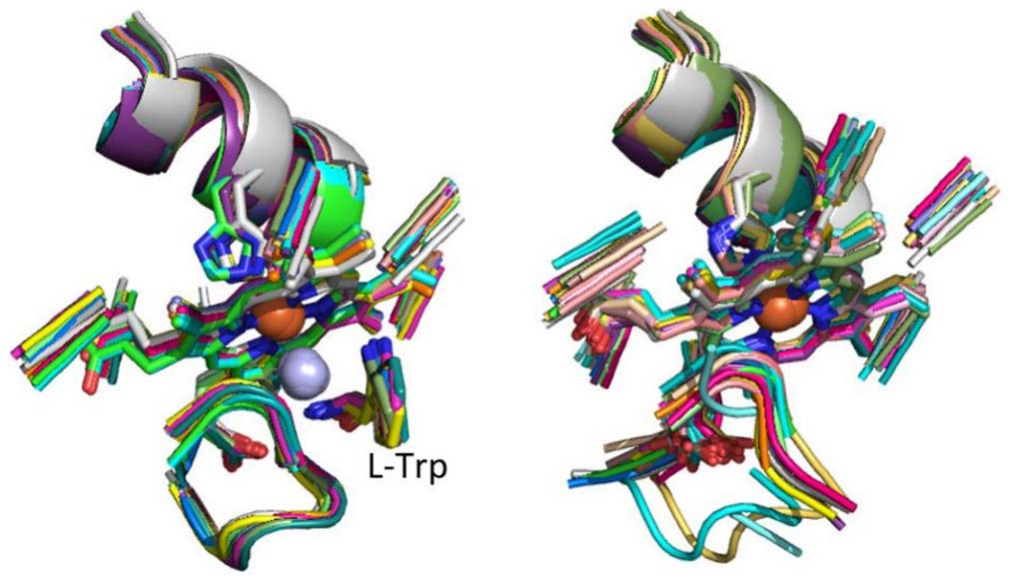
Comparison of the structures of the hMFSs contained in the two clusters originating from the equistructural group of tryptophan 2,3 dioxygenases. In the most populated cluster a molecule of substrate (L-tryptophan) is contained in the enzyme cavity (left).

For heme-containing MFSs (hMFSs hereafter), we started from 187 EGs that had more than one member. The procedure yielded 344 clusters, of which 17 clusters did not contain hMFSs and thus were discarded. Our approach readily separated sites that bind individual metal ions from hMFSs, such as in the case of the EG containing the sites corresponding to the interfacial heme of bacterioferritins. This EG additionally includes various, possibly adventitious, sites from Dps-like proteins binding cations such as iron(II), copper(II), nickel(II). The complete separation achieved upon structural comparison of these two kinds of sites is not surprising given the difference in size and interactions with the protein of the cofactor. On the other hand, the Fe-coproporphyrin III site of *Desulfovibrio desulfuricans* bacterioferritin was clustered together with all other bacterioferritin hMFSs, in keeping with its structural and functional similarity to the typical heme site^24^. Another example is that of the separation of the interfacial hMFS of *Haemophilus ducreyi* superoxide dismutase^25^ from adventitious metal sites in other superoxide dismutase structures. The 327 clusters that contained hMFSs (or other MFS binding heme analogs such as metal-substituted protoporphyrin IX, Supplementary Table S1) included 21 clusters with at least 100 sites, whereas 23 clusters contained a single hMFS. We manually inspected how the larger EGs were split into clusters. Typically, the clustering reflected defined structural features of the hMFSs. For example, in the EG corresponding to animal heme-dependent peroxidases, the two major clusters, which cumulatively accounted for 96% of the EG sites, contained myeloperoxidases together with lactoperoxidases (92 hMFSs), and prostaglandin synthases (113 hMFSs) (Figure 1). Another example is given by tryptophan 2,3 dioxygenases, which formed two clusters (18 and 23 members respectively) differing for the presence or absence of the substrate bound in the cavity (with one exception, Figure 2). In a few instances the clustering procedure generated an apparently too fine-grained separation of hMFSs. For example, the EG of cytochrome P450s, which contains 992 members, was split in as many as 22 clusters, containing 8 to 151 hMFSs. Here it was difficult to rationalize the outcome of the procedure as well as to correlate it to specific structural features. Notably, EGs including even more than 100 hMFSs constituted a single cluster when the structural similarity of the sites was sufficiently high; this was, for example, the case of the 531 hMFSs of mammalian nitric oxide synthases.

**Figure 3:**
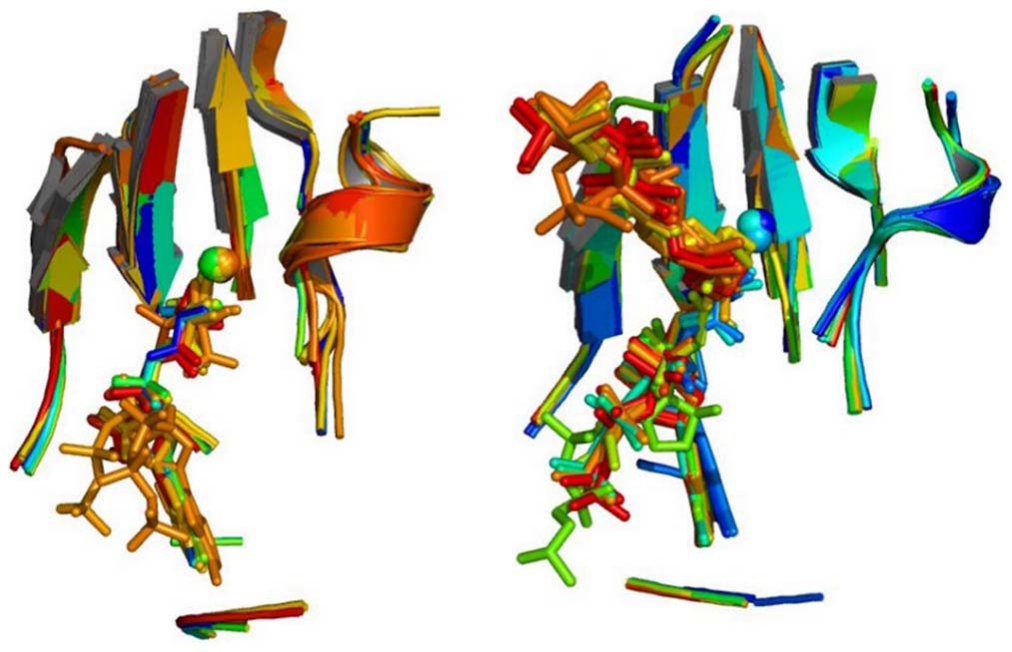
Comparison of the structures of the zMFSs contained in the two largest clusters originating from the equistructural group of aminoacyl-tRNA synthetases and closely related enzymes. The zMFSs in the two clusters differ because of the size and binding mode of their organic ligands.

For zinc-binding MFSs (zMFSs hereafter), we started from 1752 EGs with more than one member (for a total of about 19,500 zMFSs) and obtained 2263 clusters. In addition, 1640 zMFSs did not belong to any EG, and were carried on directly to the second stage of the procedure. 19 first-stage clusters included 100 sites or more. The largest cluster comprised all 335 zMFSs of the EG of alcohol dehydrogenases. As described above for hMFSs, in several cases EGs were split into two or more clusters. An interesting example is that of an EG containing 61 zMFSs from various aminoacyl-tRNA synthetases and closely related enzymes, which gave rise to four distinct clusters. Among these, the two larger clusters contained respectively 28 and 29 sites, differing for the size and binding mode of the substrate analogues present in the structure (Figure 3).

We quantified the structural deviation within clusters by computing the root-mean-square-deviation (RMSD) of the Cα and Cβ atoms of the sites. We observed that the largest average RMSD within a cluster was of only 1.5 Å. Nearly 95% of the clusters had an average RMSD smaller than 1.0 Å and the median value for the average RMSD was 0.75 Å. The very high degree of structural similarity within clusters supports the usefulness of defining a single representative MFS for each of them.

### Comparison of representative MFSs (second stage)

In the second stage of our procedure we compared representative MFSs to one another, independently of EG assignments, thus avoiding possible biases due to domain assignments. We tried different clustering approaches (complete vs. average linkage) and different thresholds (T) to evaluate the stability of the outcome (note that a higher threshold indicates lower similarity). Depending on the above factors, representatives hMFSs were grouped in a number of clusters ranging from 51 (average linkage clustering, T = 3.5) to 199 (complete linkage clustering, T = 2.25), whereas zMFSs were grouped in a number of clusters ranging from 840 (average linkage clustering, T = 2.75) to 1661 (complete linkage clustering, T = 2.25). Hereafter, we will use the following notation: CC or AC to indicate complete vs. average linkage, respectively, followed by the value of the threshold used (e.g. CC2.75 is the result of the clustering of representative hMFSs using complete linkage clustering and T = 2.75).

At the second stage of the computational procedure, there are three possible causes for representative MFSs to get clustered. (i) The first reason is that sites with very high structural similarity and found in different metalloprotein families are identified. (ii) The second cause becomes relevant when MetalPDB did not group metalloproteins of the same family, typically because of missing domain information, and consequently assigned them to different EGs. In this case, our second stage analysis puts together sites that should have been clustered already at the first stage, but actually were not compared because of the inconsistent EG assignments. (iii) The MFSs representing two clusters originating from the same EG may be regrouped because the distance between a pair of representative MFSs is shorter than the distance assigned by the CC algorithm to the corresponding clusters, as the latter equals the *largest* distance between any possible pair of cluster members. The representative MFS approximates a “central” position within the cluster it represents. This effectively reduces the distance between first stage clusters. It is possible to draw an analogy here to the use of consensus sequences to represent multiple sequence alignments, which hides some of the existing diversity. The aforementioned three causes may simultaneously concur to the formation of a second stage cluster of representative MFSs. The first and third causes should become more and more effective with reduced stringency of the clustering approach applied, whereas the relevance of the second cause is limited by the number of incomplete EG assignments and presumably declines, in relative terms, with increasing threshold.

The most stringent CC2.25 approach, which is the same approach implemented for the first stage clustering, yielded a total of 199 clusters out of 389 input hMFSs (327 representative hMFSs plus 62 singletons), each containing between 1 and 9 hMFSs. The largest clusters were formed by representative hMFSs belonging to the same EG that were re-grouped (reason iii), e.g. for some, but not all, representatives of cytochrome P450s. The representative hMFSs of tryptophan 2,3 dioxygenases (Figure 2) were also clustered together; in addition, the same cluster included the representative hMFS of the related indoleamine 2,3-dioxygenase. Example of clusters formed only at the second stage because of missing domain assignments (reason ii) in MetalPDB were that of the sirohemes in the catalytic sites of sulfite reductases, or of dye peroxidases (DyP). For the latter case, the appropriate domain is not identified within the sequence of DyP2 from *Amycolatopsis* sp. ATCC 39116 (PDB entry 4G2C^26^) but our approach correctly identified the similarity between DyP hMFSs. Finally, the cluster containing heme 4 of the cytochrome *c* subunit of *Rhodopseudomonas viridis* photosyntethic reaction center and the cysteine-coordinated heme of SoxA (heme 1263 in the 1H32 structure^27^) is an example of a cluster formed with CC2.25 for reason (i), i.e. because highly similar hMFS occurred in proteins with unrelated fold. With increasing threshold or passing from the CC to the AC approach, the number of clusters diminished as the reduced stringency allowed more dissimilar sites to be clustered together than for CC2.25. In particular, 110 clusters were formed with AC2.75 (Supplementary Table S2). We previously showed that 2.75 is a reasonable threshold for the MetalS^2^ score^10^ to identify meaningful structural similarities. At this level all cytochrome P450s were clustered together but one (PDB entry 3R9C^28^), due to the presence of a sodium(I) ion within the latter hMFS. Other metalloprotein families remained split even at this level, such as the family of ABM monooxygenases, which include various heme-degrading enzymes, reflecting their different modes or stoichiometries of heme binding^29^. When applying the AC2.75 approach, clusters formed with CC2.25 can merge. This occurred, for example, for the aforementioned cluster of tryptophan and indoleamine 2,3-dioxygenases, which additionally included the heme site of proteins related to PnrB, the second enzyme in the pyrrolnitrin biosynthesis pathway. Thus, our approach recomposed the full group of related dioxygenase folds, which eventually comprised proteins from three different EGs of MetalPDB.

**Figure 4:**
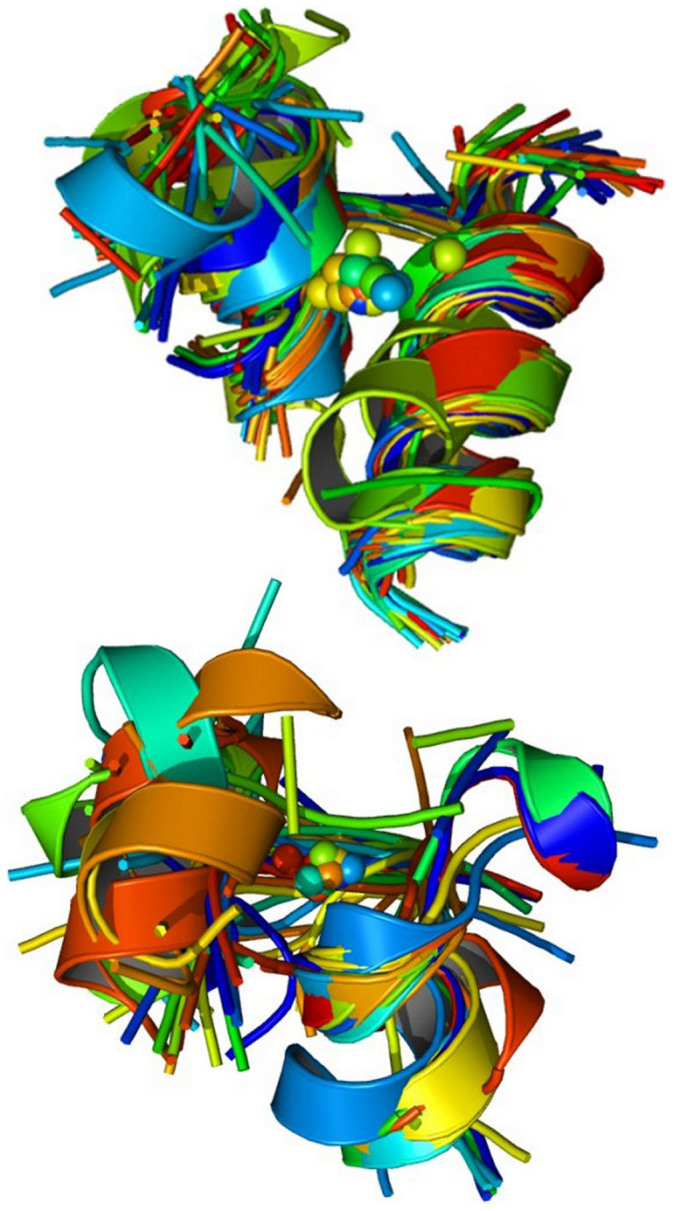
Example clusters of representative zMFSs. (Top) superposition of 66 representative zMFSs of different related metallopeptidases; the common position for substrate binding, as indicated by the binding of ligands (hidden for clarity) in the 3D structures of the cluster, faces the reader; (Bottom) superposition of 31 representative zMFSs of non-standard zinc fingers.

**Figure 5:**
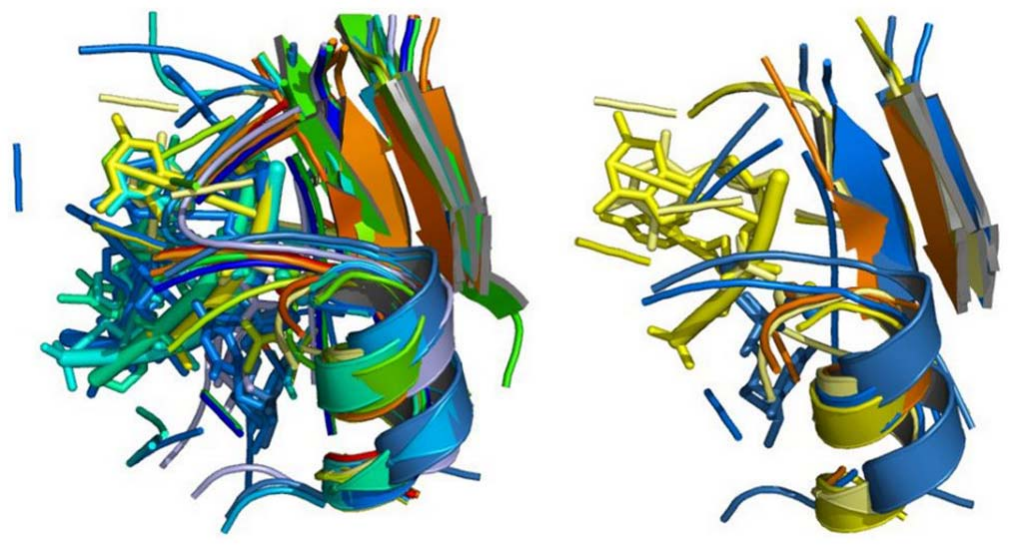
A cluster formed by zMFS from DNA polymerases with nuclease activity and 2-C-methyl-D-erythritol 2,4-cyclodiphosphate synthases. The ligands or substrates present in the structures are also shown. The right panel depicts a selection of two synthases (blue structures), two exonuclease sites of polymerases (yellow structures) and of the fission yeast Pop2p exonuclease (orange).

For zMFSs, we analyzed in detail the output of the AC2.5 clustering, which provided 1083 clusters (of which 763 with more than one member; Supplementary Table S3). Our analysis focused on the ten largest clusters, which ranged in size between 25 and 382 members. The superpositions corresponding to two of these clusters are shown in Figure 4. In the top panel, a single cluster encompasses 66 representative zMFSs of different types of related peptidases, largely from the metallopeptidase MA clan^30^. It further includes the active sites of the anthrax toxin lethal factor^31^ and, curiously, the zinc-substituted catalytic site of iron-dependent tyrosine 3-monooxygenase (PDB ID 2XSN, unpublished). The superposition clearly reveals that the local structural similarity extends to the region of substrate binding. The bottom panel of Figure 4 instead refers to a 31-member cluster, which mainly includes zinc-finger-type sites from a variety of systems. These zMFSs are identified in proteins from prokaryotic as well as eukaryotic organisms and their functional role has not always been ascertained. Whereas interaction with DNA seems the most obvious role^32^, also because the majority of these systems are involved in DNA recognition and/or modification and repair, there are other possibilities, such as ubiquitin-binding^33^. In previous articles, these zMFS have been described as unique to a specific system^34^ or not relevant to function^35^. Instead, the present data show that it is relatively widespread and thus likely to have functional relevance. This highlights the usefulness of the present approach as a knowledge discovery tool in bioinorganic chemistry. Finally, the cluster shown in Figure 5 contains 25 zMFSs from enzymes, mostly di-nuclear metal sites formed by zinc(II) and another divalent cation. The zinc ion is the catalytic center of these enzymes, whereas the second metal ion might be bound to the substrate (e.g. Mg- cytidine diphosphate for 2C-methyl-d-erythritol-2,4-cyclodiphosphate synthase^36^) or can be bound to the protein independently of the presence of substrate/cofactors (e.g. Mn(II) in yeast Pop2p^37^). The site is found either in 2C-methyl-d-erythritol-2,4-cyclodiphosphate synthases or in DNA polymerases with exonuclease activity as well as other nucleases (Figure 5). These groups of enzymes share a similar architecture but different topologies, according to the CATH^38^ classification. Intriguingly, despite the different fold, the substrate binding site is closely located in these two groups. The same zMFS is exploited to perform a phosphorus-oxygen lyase reaction by the synthases, with respect to the hydrolysis of a phosphodiester bond in the nucleases.

### A detailed analysis of multiheme c-type cytochromes

**Figure 6:**
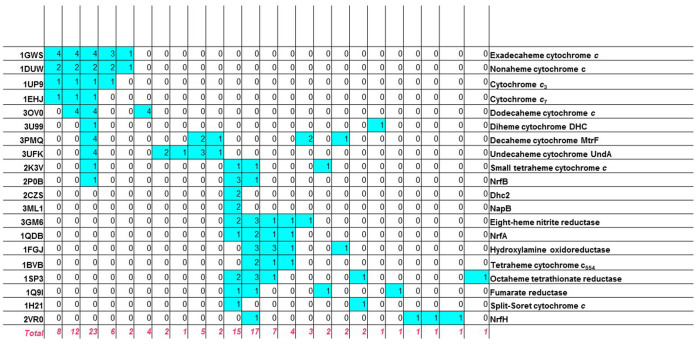
Structural relationships between the hMFSs of multi-heme cytochromes. The number of hMFSs for a given MHC (rows) included in a given cluster (columns) is reported. Each column corresponds to a cluster of Supplementary Table S2. Each row corresponds to a different MHC family. The first column reports the PDB entry corresponding to the structure of a typical member of the family (not necessarily the one from which representative hMFSs are derived). The last row reports the number of hemes in each cluster.

**Figure 7:**
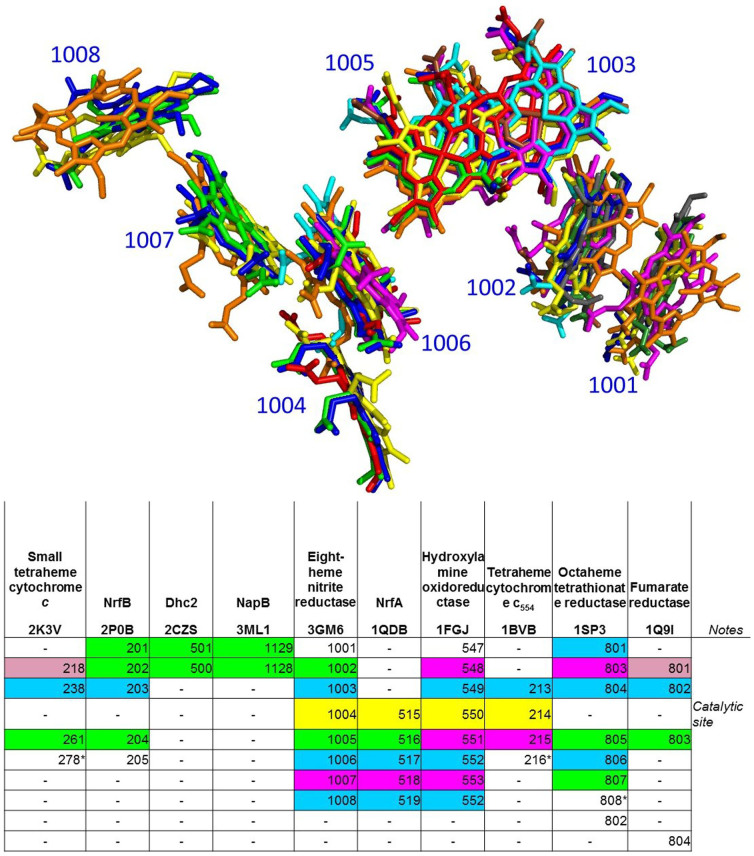
hMFS relationships in eight-heme, NrfA and related MHCs derived from cluster-guided structural superpositions. Top: Superpositions of the heme groups of selected MHCs resulting from the simultaneous overlay of the protein part of the hMFSs of each MHC to the sites of eight-heme nitrite reductase (PDB entry 3GM6) belonging to the same AC2.75 clusters (2K3V, cyan; 2P0B, magenta; 2CZS, gray; 3ML1, dark green; 3GM6, blue; 1QDB, light green; 1FGJ, yellow; 1BVB, red; 1SP3, orange; 1Q9I, brown). Residue numbering for the heme groups is shown for structure 3GM6. Bottom: summary of the relationships, color coded according to the cluster assignments of Figure 4 (green: cluster 65; blue: cluster 68; magenta: cluster 64; yellow, cluster 55; pink, cluster 61). Heme sites are labeled by their residue numbers in the PDB structure. Relationships are derived from spatial proximity after superposition and all refer to the sites of nitrite reductase. Only clusters containing hMFSs from different MHCs have been highlighted. A star indicates sites that fulfill the requirement of spatial proximity but are not satisfactorily superimposed (e.g. iron ligands do not overlay or the heme orientation is somewhat different). The heme groups 802 of structure 1SP3 and 804 of structure 1Q9I have been omitted for clarity. The figure independently re-discovers the known^43^ relationships between between hemes I–VIII of nitrite reductase and hemes I–VIII of hydroxylamine oxidase, between hemes IV–VIII of nitrite reductase and hemes I–V of NrfA, or between seven out of the eight groups of nitrite reductase and tetrathionate reductase.

Multiheme *c*-type cytochromes (MHCs), which are proteins that bind several heme groups to a single polypeptide chain via a pair of thioether bonds, are of particular interest in the context of the present work. For these systems fold assignments tend to be less informative, also because their 3D structure is largely determined by cofactor-protein hydrophobic interactions rather than by protein-protein interactions in the hydrophobic core^39^. Our protocol provided a complete picture of structural similarities among the various hMFSs contained in MHCs, from di-heme to sixteen-heme proteins (Figure 6). It is possible to immediately identify two major blocks of related MHCs, namely those linked to (or, in evolutionary terms, presumably derived from) the four hMFSs of the tetra-heme cytochrome *c*_3_ and those linked to the sites of NrfA. The first block includes cytochrome *c*_3_, cytochrome *c*_7_, nona-, dodeca- and exadeca-heme cytocromes. In the first block, all hMFSs can be related to one of the hMFS of cytochrome *c*_3_, with two exceptions. One is a unique site present in nonaheme cytochromes that acts as a connector between two cytochrome *c*_3_ domains^40^. A search of the MetalPDB database using this site as input to the MetalS^3^ search tool^6^ revealed a weak similarity to one hMFS of NrfB (not shown). The other exception was within the structure of dodecaheme cytochromes, which have been described as a combination of four cytochrome *c_7_* domains^41^. Our analysis indicated that this is true for two out of three hMFSs, whereas the other hMFS is structurally diverse and gave rise to a separate cluster (Figure 6 and Supplementary Figure S2). The second block includes NrfA (a five-heme nitrite reductase), NrfB (a five-heme electron donor to NrfA), eight-heme nitrite reductase, hydroxylamine oxidase (a eight-heme enzyme), tetrathionate reductase (a eight-heme enzyme) and tetra-heme cytochrome *c*_554_. Here all hMFSs can be related to one of the sites of NrfA, with one or two specific exceptions for NrfB as well as the various eight-heme enzymes. Furthermore, we indentified a tight relationship between the hMFSs of two of the simplest MHCs, namely the di-heme proteins NapB, a subunit of periplasmic nitrate reductase, and *Geobacter sulfurreducens* DHC2 cytochrome *c*. The analysis summarized by Figure 6 provides an objective guidance to comparison at the whole structure level for pairs of MHCs with different folds. Indeed, after superposition of the hMFSs of the two proteins contained in the same clusters MetalS^2^ provides roto-traslational matrices that can be applied to the entire structure. Cluster assignments indicate how to combine various hMFSs to obtain a single overall matrix that yields a best fit for all of them simultaneously. The global structural superposition obtained in this way can indicate relationships also between sites not clustered together, based on the spatial proximity of the heme groups (Supplementary Figure S3). As an example, Figure 7 provides an overview of the hMFS correspondences obtained by superposing various MHCs to the structure of eight-heme nitrite reductase (PDB entry 3GM6^42^) as indicated above. The known^43^ relationships between the sites of these proteins are independently re-discovered. Notably, the catalytic sites of nitrite reductase, hydroxylamine oxidase, NrfA and cytochrome *c*_554_ are related by spatial proximity after superposition in addition to their belonging to the same cluster. For cytochrome *c*_554_, a NO reducing activity has been reported^44^; its structural correspondence to hydroxylamine oxidase, including the then unknown catalytic site, had already been highlighted^45^. A less obvious relationship is that between three sites of fumarate reductase and three sites of the small tetraheme cytochrome *c* from S*hewanella*. (Figure 7).

## Discussion

In this work, we developed a methodology to perform a systematic comparison based on structural similarity of metal sites extracted from metalloproteins. Our definition of metal site extended beyond the metal ion and its aminoacidic ligands by involving all the chemical species (aminoacids, nucleotides, exogenous ligands) containing at least one donor atom (shown in blue in Supplementary Figure S1) as well as all any other chemical species within a radius of 5.0 Å (shown in green in Supplementary Figure S1). We previously defined this as the minimal functional site of a metalloprotein (MFS), and showed that its characteristics are related to the metalloprotein function^3^,^7^. The present methodology leverages the MetalS^2^ algorithm, whose total score provides a quantitative measure of structural similarity between pairs of MFSs^10^. We used this measure to build clusters of structurally similar MFSs using a hierarchical clustering algorithm. The proposed computational strategy is a two-stage procedure, mainly for the sake of simplicity and calculation speed. In the first stage, predefined groups of MFSs contained in corresponding regions of metalloproteins having the same fold (equistructural groups, EGs) are retrieved from the MetalPDB database^9^. Then, all MFSs in each EG are systematically compared to one another. After the application of a complete linkage clustering algorithm with a very restrictive threshold (2.25) each EG gave rise to one or more clusters characterized by a low degree of internal structural variability (less than 1 Å backbone RMSD in more than 90% of the cases). The different clusters resulting from a given EG provide a thorough view of homogeneous structural features across the members of the group. Because each EG corresponds to a specific metalloprotein family, the first stage clusters recapitulate systematically the known structural variants of the metal-binding site of that family. These variants can be associated to biochemical events such as ligand binding (Figure 2 and Figure 3) or reflect the structural features of different subfamilies (Figure 1). The low structural variability within clusters enabled us to meaningfully define a single representative MFS for each cluster.

Representative MFSs allow the comparison of the sites of different metalloprotein families (second stage clustering), at the level of their structural subtype, in an innovative manner that is independent of the global sequence or structural similarity of the metalloproteins containing the MFSs. Indeed, the clusters obtained after the second stage often grouped MFSs from metalloproteins with different but related folds (e.g. as defined by so-called clans in the Pfam database of domains^46^). This supports the idea that the 3D structures of the whole metalloprotein and of its metal site differentiate at comparable rates. The detection of structural similarity between MFSs can thus be taken as good an indication of homology as overall structural similarity is for proteins not binding metal cofactors. This result provides also a means to assign potential biological functions to the so-called domains of unknown function, when they contain MFSs structurally similar to sites of functionally characterized metalloproteins. Finally, discovering structural similarities among representative MFSs also allows establishing relationships involving completely unrelated protein domains.

We demonstrated a practical implementation of the proposed procedure for heme-binding proteins as well as for zinc-binding proteins. The unique usefulness of the present tool resides in its capability to address comprehensively relationships among different metalloprotein families, i.e. in systems with different folds. As observed for MHCs, such relationships can be related to evolutionary patterns (Figure 6) but can also correct or shed a different light on previously proposed such patterns (Supplementary Figure S2). Furthermore, our approach identified common occurrences of zinc-binding sites across different protein folds, showing how the same local structure is harnessed by different systems to perform different metal-based catalysis (Figure 5).

In conclusion, we showed here for the first time that the structures of MFS, i.e. of small portions of the larger 3D structures of metalloproteins and metalloenzymes centered around the metal cofactor, can be systematically compared and clustered to obtain useful insight into the structural, functional and evolutionary features of metalloproteins. This kind of analysis complements the information that can be gained through more conventional approaches, such as sequence or fold comparison^13^,^14^,^15^,^16^,^17^,^18^. The present protocol constitutes a unique, innovative tool in the portfolio of computational tools of bioinorganic chemists. Its unicity stems from the concept of centering structural comparisons at the metal center itself, which is crucial to define the cellular role of metal-binding proteins. By performing comparisons at the level of the whole MetalPDB database, users can achieve a systematic view of metalloproteins based on the structural properties of the metal sites rather than on the structural properties of the protein fold in which the site is embedded, as afforded by currently available approaches. This is a dramatically different viewpoint on metalloproteins, which only now becomes available.

## Methods

### Background

In our previous work^9^ we organized MFSs into groups of equistructural sites. Such sites are extracted from metal-binding polypeptide chains that have similar fold, using the approach summarized below. After superimposing all the chains with the same fold, the distance between the metal ions (or the geometric center of all metal ions for polymetallic cofactors) is measured. MFSs whose metal ions are separated by a distance shorter than a predefined threshold (3.5 Å) are put in the same group, regardless of the chemical identity of the ions. This leads to e.g. all sites of the same metalloprotein after different metal replacement experiments belonging to the same equistructural group. Broadly speaking, the condition described above identifies sites that occupy the same location within a given protein fold. At the computational level, a single linkage clustering approach has been implemented to build the groups. A practical implication of this is that for any given MFS in a group the aforementioned condition will be fulfilled by at least another group member, but not necessarily by all. By construction, the structural similarity that is described by equistructural groups is mainly the result of overall fold similarity. Conversely, structurally similar MFSs that are bound to proteins with different fold were associated with different equistructural groups. Here, we combine the use of our MetalS^2^ algorithm, which provides a quantitative approach to the structural comparison of pairs of MFSs^10^, with a hierarchical clustering method to cluster MFS structures independently of the overall metalloprotein fold.

### Datasets used

The datasets used for this study consist of the three-dimensional structures of all MFSs present in the MetalPDB database (http://metalweb.cerm.unifi.it/) as of April 2014 that were members of an equistructural group containing at least one heme-binding site or at least one zinc ion.

The number of heme sites in the dataset was 8891, separated into 249 EGs. Of these, 14 contain at least 100 members, with the largest one having more than 2000, whereas 62 are singletons, i.e. contain only one site. To achieve the greatest coverage and potentially gain more information, the above included also sites that harbor chemically or biosynthetically modified heme cofactors as well as inorganic complexes mimicking the heme moiety (Supplementary Table S1).

For zinc-binding sites, we firstly removed all sites with less than 10 amino acids as well as all sites where the zinc ion had only one aminoacidic ligand will all other ligands being water molecules. The number of zinc sites was 21483, of which we kept 20478. After the first stage, we obtained 2263 clusters, plus 1640 singletons.

### Clustering procedure

Our procedure was based on a hierarchical agglomerative clustering algorithm^47^. In agglomerative clustering every individual object is initially considered as a singleton (i.e. a cluster containing only one member). Then the clusters are iteratively grouped by merging the two clusters at the shortest distance, i.e. the most similar pair. For the present work, the operative distance measure adopted was the global MetalS^2^ score, which increases with increasing structural diversity^10^. Two merged clusters become one cluster, so after each iteration there is one less cluster. The iterations are repeated until all objects are collected into a single cluster. The result of hierarchical clustering is a nested sequence of partitions, with a single, all inclusive cluster at the top and singleton clusters at the bottom. Each intermediate cluster can be viewed as a combination of two clusters from the lower level or as a part of a split cluster from the higher level. Hierarchical clustering methods differ in the way they merge clusters. Although all methods merge the two “closest” clusters at each step, they determine differently the distance between clusters, i.e. have different metrics to compare one cluster to another. We used the complete and average linkage methods. For complete linkage the distance between a pair of clusters corresponds to greatest distance from any member of one cluster to any member of the other cluster. In other words, the distance between clusters *C_i_* and *C_j_* is defined as
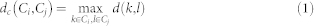


In the average linkage method the distance between two clusters is the average of the distances between all the members in one cluster and all the members in the other. The distance for the average linkage is defined as
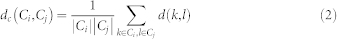
where |*C_i_*| and |*C_j_*| are the numbers of members in the clusters C_i_ and C_j_ correspondingly.

In both formulas *k* and *l* refer to members of the clusters C_i_ and C_j_, *d(k,l)* is the distance between the *k*-th member and *l*-th member of, respectively, C_i_ and C_j_ (in practice the global MetalS^2^ score between the *k*-th and *l*-th MFSs). The minimum distance *d_c_(C_i_,C_j_)* among all the intra-cluster distances determines which pair of clusters is merged.

The clustering results are influenced by the linkage type applied. Complete linkage tends to produce clusters that are more compact (tight) with respect to clusters produced by average linkage. When a cut-off value of a similarity measure is applied in order to determine the final partition, the clusters produced by the average linkage method allows some within-cluster distances to exceed the cut-off value whereas the complete linkage method ensures that no within-cluster distance exceeds the cut-off. As a result, the complete linkage approach produces a higher number of more robust clusters while with average linkage the number of clusters is lower but within-cluster variability is higher. One of the weaknesses of the complete linkage method is its sensitivity to outliers, i.e. members that do not fit well into the global structure of the cluster. Such sensitivity may prevent the identification of even intuitive clusters, as outliers may pull similar members into different groups.

For the analysis of our dataset, we used the algorithm described above within a multi-step procedure, which included: (i) dividing existing equistructural groups into smaller clusters (first, intra-group stage); (ii) defining a representative MFS for each cluster; (iii) building broader clusters by comparing the representative MFSs from clusters built at the first level (second, inter-group stage).

### First stage

This stage of analysis is designed to capture the structural variations possibly occurring among the MFSs in each group of equistructural sites of MetalPDB. For each group we systematically compared all possible pairs of MFSs, using the MetalS^2^ algorithm^10^. The result was a matrix of all-versus-all comparison scores for each group. The matrix was then used as the input to perform hierarchical clustering within the equistructural group, applying a cut-off value of 2.25 for the MetalS^2^ similarity score to build the clusters. At this stage we applied a complete linkage clustering approach, so that two MFSs whose structural superposition results in a MetalS^2^ score greater than 2.25 are always associated to different clusters. As we described previously^10^, the 2.25 threshold is quite stringent, i.e. corresponds to a high level of structural similarity. The first-stage procedure thus resulted in a fine-grained clustering of each EG, which highlighted intra-group structural variations.

For each cluster obtained that contained more than one MFS, we defined a single representative MFS as the most similar on average to all other members of the cluster. In practice the representative MFS was defined as the MFS minimizing the sum of the MetalS^2^ global scores resulting from its pairwise comparisons to all other MFSs in the same cluster. All the MFSs that did not cluster at the first stage or that formed an equistructural group (singleton) by themselves were taken as a representative. Clusters that did not contain heme-binding sites were removed, together with their corresponding representatives.

### Second stage

The second stage of comparison aims to obtain a set of clusters, each representing a distinct MFS shape, independently of overall protein fold. The dataset used for this analysis included all representative structures of MFSs from the first level clustering. Similarly to the first stage procedure, we generated a single all-versus-all similarity matrix. Both complete and average linkage clustering algorithms were then applied to generate clusters at this stage. Different cut-off values, from 2.25 to 3.5, were tested.

### Multi-heme c-type cytochromes

To investigate the full network of structural relationships across multi-heme *c*-type cytochromes (MHCs) we compiled a list of all clusters that included sites from different MHCs (we excluded MHCs containing multiple single-heme mitochondrial-type cytochrome c domains^14^). For each protein we then added the clusters containing only hMFSs specific to it in order to cover its entire set of sites (either common to other MHCs or unique to that MHC). This was done for all proteins in the MHC list, so that the set of selected clusters eventually contained all MHC sites present in the MetalPDB database.

## Supplementary Material

Supplementary Information Supplementary Figures and Table S1

Supplementary Information Table S2

Supplementary Information Table S3
