# Supplementary material for: Hidden relationships between metalloproteins unveiled by structural comparison of their metal sites
Source: Sci Rep. 2015 Mar 30;5:9486. doi: 10.1038/srep09486 (PMC4377587; doi:10.1038/srep09486)
Supplement: Supplementary Information — Supplementary Figures and Table S1 [file srep09486-s1.doc]

# Hidden relationships between metalloproteins unveiled

# by structural comparison of their metal sites

Yana Valasatava^1^, Claudia Andreini^1, 2^, and Antonio Rosato^1, 2, *^

^1^Magnetic Resonance Center (CERM) – University of Florence, Via L. Sacconi 6, 50019 Sesto Fiorentino, Italy

^2^Department of Chemistry – University of Florence, Via della Lastruccia 3, 50019 Sesto Fiorentino, Italy

**SUPPLEMENTARY INFORMATION**

**Supplementary Figure S1. Example of a heme-binding MFS**

**Supplementary Figure S2. Structural superimposition of the hMFSs of one domain of twelve-heme cytochtrome c to the corresponding hMFSs of cytochrome c7, highlighting the structural divergence of one of the three hMFSs.**

**Supplementary Figure S3. Example of a the superposition of two MHC structures based on the superposition of the backbone of pairs of hMFSs assigned to the same clusters.**

**Supplementary Table S1. List of all heme-containing and heme-related cofactors present in the MFS analyzed in this work**

**Supplementary Table S2. List of all hMFS clusters generated at the second stage of the protocol with AC2.75**

**Supplementary Table S3. List of all zMFS clusters generated at the second stage of the protocol with AC2.5**

**Supplementary Figure S1. Example of a heme-binding MFS**


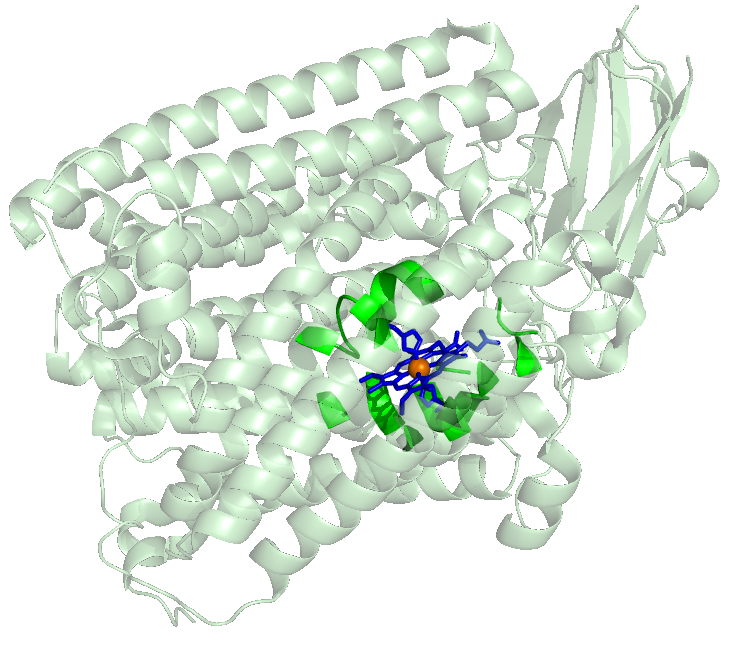


**Supplementary** **Figure S2. Structural superimposition of the hMFSs of one domain of twelve-heme cytochtrome *c* to the corresponding hMFSs of cytochrome *c_7_*, highlighting the structural divergence of one of the three hMFSs**.


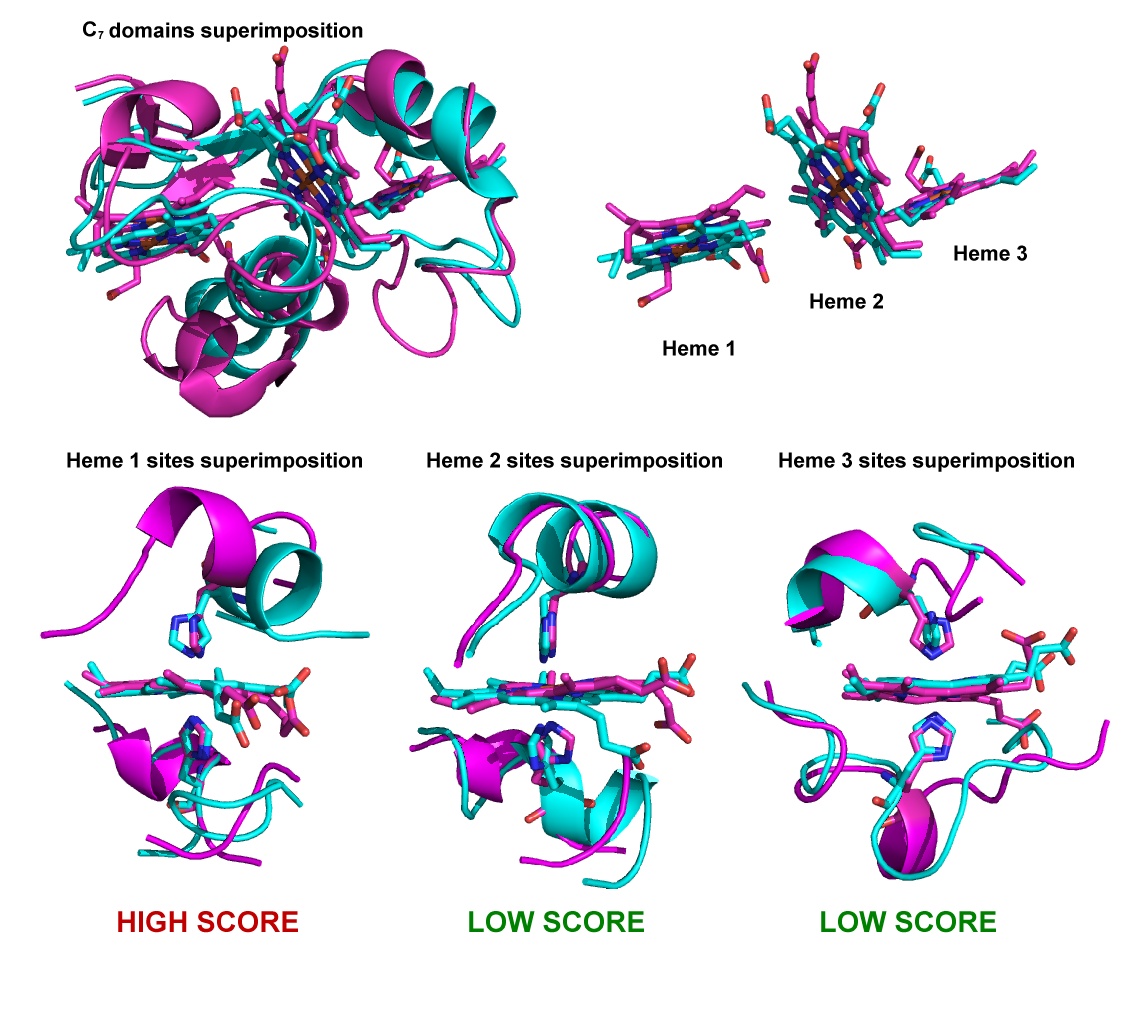


**Supplementary Figure S3. Example of the superposition of two MHC structures based on the superposition of the backbone of pairs of hMFSs assigned to the same clusters.** Red: tetraheme cytochrome *c554* (PDB entry 1BVB); blue: eight-heme nitrite reductase (PDB entry 3GM6). A) superposition of the two hMFS pairs clustered together (Figure 6); B) view of all heme groups in the superposition, showing that hemes 1005 and 1006 of 3GM6 and are additionally put in relation with hemes 215 and 216 of 1BVB. The numbering of heme groups is the same as in the corresponding PDB entries.


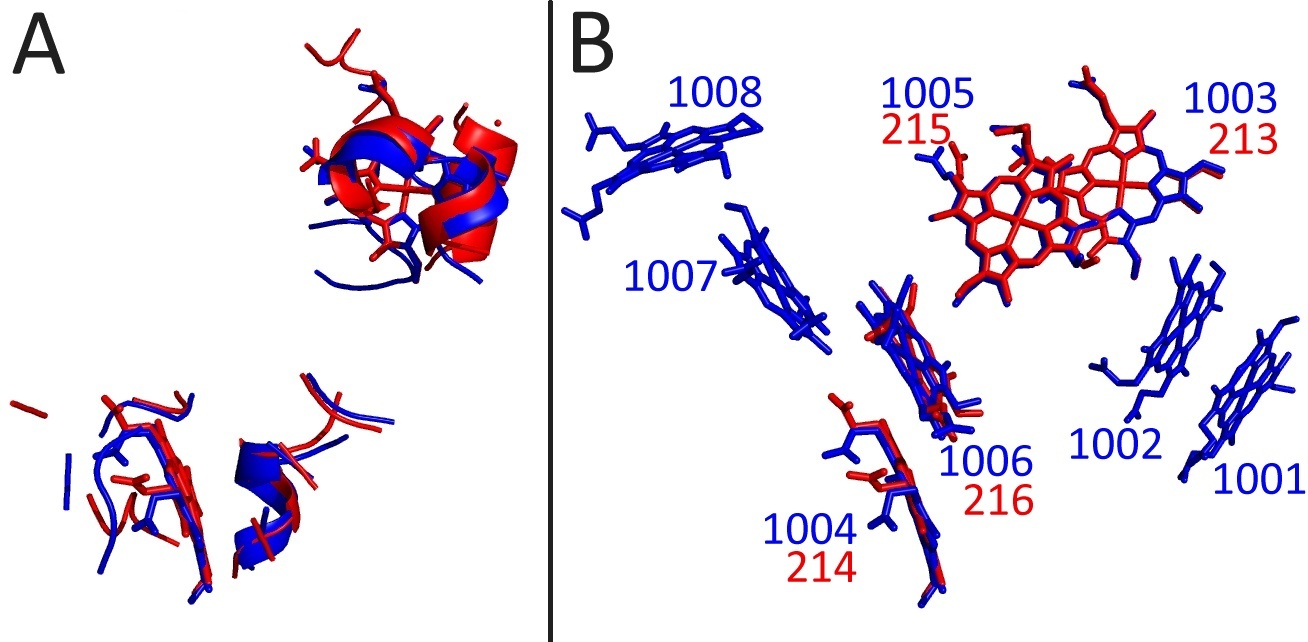


Supplementary Table S1. List of all heme-containing and heme-related cofactors present in the MFS analyzed in this work

| **PDB CODE** | **EXTENDED NAME** |
| --- | --- |
| HEM | PROTOPORPHYRIN IX CONTAINING FE |
| HEC | HEME C |
| DHE | HEME D |
| SRM | SIROHEME |
| HEA | HEME-A |
| CCH | [7-ETHENYL-12-FORMYL-3,8,13,17-TERTRAMETHYL-21H,23H-PORPHINE-2,18-DIPROPANOATO(2)-N21,N22,N23,N24]IRON |
| HAS | HEME-AS |
| HEV | 5,8-DIMETHYL-1,2,3,4-TETRAVINYLPORPHINE-6,7-DIPROPIONIC ACID FERROUS COMPLEX |
| HEO | HEME O |
| HDD | CIS-HEME D HYDROXYCHLORIN GAMMA-SPIROLACTONE |
| HE6 | 6,7-DICARBOXYL-1,2,3,4,5,8-HEXAMETHYLHEMIN |
| HIF | FE(III)-(4-MESOPORPHYRINONE) |
| HDM | DIMETHYL PROPIONATE ESTER HEME |
| FEC | 1,3,5,8-TETRAMETHYL-PORPHINE-2,4,6,7-TETRAPROPIONIC ACID FERROUS COMPLEX |
| HEB | HEME B/C |
| HCO | 2-ACETYL-PROTOPORPHYRIN IX |
| HFM | 2-FORMYL-PROTOPORPHRYN IX |
| 2FH | 2-PHENYLHEME |
| FMI | FE-(4-MESOPORPHYRINONE)-R-ISOMER |
| 1FH | 12-PHENYLHEME |
| VER | IRON-OCTAETHYLPORPHYRIN |
| CLN | SULFUR SUBSTITUTED PROTOPORPHYRIN IX |
| FDE | FE(III) DEUTEROPORPHYRIN IX |
| FDD | FE(III) 2,4-DIMETHYL DEUTEROPORPHYRIN IX |
| POR | PORPHYRIN FE(III) |
| HME | PORPHYCENE CONTAINING FE |
| 6HE | 6-METHY-6-DEPROPIONATEHEMIN |
| 7HE | 7-METHYL-7-DEPROPIONATEHEMIN |
| VEA | 5-OXA-PROTOPORPHYRIN IX CONTAINING FE |
| HKL | FE(III) PYROPHEOPHORBIDE-A METHYL ESTER |
| NTE | [3,3'-{7-ETHENYL-3,8,13,17-TETRAMETHYL-12-[(E)-2-NITROETHENYL]PORPHYRIN-2,18-DIYL-KAPPA~4~N~21~,N~22~,N~23~,N~24~}DIPROPANOATO(2-)]IRON |
| HDE | CIS-HEME D HYDROXYCHLORIN GAMMA-SPIROLACTONE 17R, 18S |
| MH0 | MESOHEME |

**Supplementary Table S2. List of all hMFS clusters generated at the second stage of the protocol with AC2.75**

Attached Excel file

**Supplementary Table S3. List of all zMFS clusters generated at the second stage of the protocol with AC2.5**

Attached Excel file
